# Arsenic Exposure Affects Plasma Insulin-Like Growth Factor 1 (IGF-1) in Children in Rural Bangladesh

**DOI:** 10.1371/journal.pone.0081530

**Published:** 2013-11-26

**Authors:** Sultan Ahmed, Rokeya Sultana Rekha, Khalid Bin Ahsan, Mariko Doi, Margaretha Grandér, Anjan Kumar Roy, Eva-Charlotte Ekström, Yukiko Wagatsuma, Marie Vahter, Rubhana Raqib

**Affiliations:** 1 Centre for Vaccine Sciences, International Centre for Diarrhoeal Disease Research, Bangladesh (ICDDR,B), Dhaka, Bangladesh; 2 Institute of Environmental Medicine (IMM), Karolinska Institutet, Stockholm, Sweden; 3 Department of Clinical Trial and Clinical Epidemiology, Faculty of Medicine, University of Tsukuba, Tsukuba, Japan; 4 International Maternal and Child Health, Department of Women’s and Children’s Health, Uppsala University, Uppsala, Sweden; Inserm, France

## Abstract

**Background:**

Exposure to inorganic arsenic (As) through drinking water during pregnancy is associated with lower birth size and child growth. The aim of the study was to assess the effects of As exposure on child growth parameters to evaluate causal associations.

**Methodology/Findings:**

Children born in a longitudinal mother-child cohort in rural Bangladesh were studied at 4.5 years (n=640) as well as at birth (n=134). Exposure to arsenic was assessed by concurrent and prenatal (maternal) urinary concentrations of arsenic metabolites (U-As). Associations with plasma concentrations of insulin-like growth factor 1 (IGF-1), calcium (Ca), vitamin D (Vit-D), bone-specific alkaline phosphatase (B-ALP), intact parathyroid hormone (iPTH), and phosphate (PO_4_) were evaluated by linear regression analysis, adjusted for socioeconomic factor, parity and child sex. Child U-As (per 10 µg/L) was significantly inversely associated with concurrent plasma IGF-1 (β=-0.27; 95% confidence interval: -0.50, -0.0042) at 4.5 years. The effect was more obvious in girls (β=-0.29; -0.59, 0.021) than in boys, and particularly in girls with adequate height (β=-0.491; -0.97, -0.02) or weight (β=-0.47; 0.97, 0.01). Maternal U-As was inversely associated with child IGF-1 at birth (r=-0.254, P=0.003), but not at 4.5 years. There was a tendency of positive association between U-As and plasma PO_4_ in stunted boys (β=0.27; 0.089, 0.46). When stratified by % monomethylarsonic acid (MMA, arsenic metabolite) (median split at 9.7%), a much stronger inverse association between U-As and IGF-1 in the girls (β=-0.41; -0.77, -0.03) was obtained above the median split.

**Conclusion:**

The results suggest that As-related growth impairment in children is mediated, at least partly, through suppressed IGF-1 levels.

## Introduction

Exposure to As during pregnancy has been associated with pre-term births and increased risks of fetal and infant mortality [1-4]. Elevated arsenic concentrations in drinking water have also been associated with lower birth weight [2,5]. In our ongoing population-based mother-child cohort study in rural Bangladesh, we detected lower size at birth in relation to relatively low levels of arsenic exposure during pregnancy (<100 µg/L in maternal urine) [6,7]. The inverse associations between maternal As exposure and fetal size, measured by ultrasound in early and late pregnancy, were most obvious for the head measures and femur length [6]. The effects on child growth remained until 5 years of age and were aggravated by continuous exposure after the breast-feeding period [8]{Gardner, 2013 #742}.

Arsenate (AsV) is known to accumulate in bone, due to its chemical similarities to PO_4_ [9], while trivalent arsenic has been found to inhibit cartilage formation in chick limb bud mesenchymal culture [10]. In animal studies, offspring exposed to high arsenic doses *in utero* had reduced fetal weight and increased frequency of axial skeletal malformations [11,12], effects that were accentuated by protein deficiency [13]. Furthermore, experimental animals exposed to As in drinking water showed alteration in endochondral ossification during bone remodeling [14]. 

In this study, we aimed to elucidate the mechanisms involved in arsenic-related fetal and child growth impairment, using biochemical indicators of bone development and growth. Specifically for growth, we assessed the IGF-1 hormone, which is a crucial mediator of body size, embryonic and postnatal development, and affects skeletal muscle, cartilage and bone. We hypothesized that in addition to the concurrent exposure, prenatal exposure would contribute to an accumulating effect of continued As exposure on child growth. Studying the prenatal and concurrent arsenic exposure would thus allow deciphering the early and later effects of As on the biomarkers of growth.

## Results

### Demographic data and As exposure

The average height and weight of the children at 4.5 years of age were 100 cm and 14.0 kg respectively with a male to female ratio of 51:49 (Table 1). Among the children with low birth weight (< 2500 g), 61 were boys and 89 girls. There were more stunted and underweight girls than boys (P=0.035 and 0.045 respectively). In the prenatal cohort, the average age of pregnant women (n=134) was 25.6 years at enrolment and the average gestational age at delivery was 39.3 weeks (Table 1). Of the 134 births, 25 infants (13 boys and 12 girls) were of low birth weight. All women were non-smokers; however, 7% of the women reported chewing tobacco. The median U-As in mothers at gestational week 8 (GW8) was 78 µg/L and at GW30 was 71 µg/L and in 4.5 year old children it was slightly lower, 57 µg/L (Table 1).

**Table 1:** Characteristics of the study cohorts.

**Variables**	Child cohort (N=640)	Prenatal cohort (N=134)
**4.5 years old children**		
Height, cm	100 ± 4	
Weight, kg	13.9 ± 1.6	
HAZ	-1.57 (-2.98; -0.11)	
Stunted, n (%)	185 (29)	
Stunted Girls, n (%)	102 (33)	
Stunted Boys, n (%)	83 (26)	
WAZ	-1.80 (-3.08; -0.42)	
Underweight, n (%)	252 (39)	
Underweight Girls, n (%)	135 (43)	
Underweight Boys, n (%)	117 (36)	
Urinary As, µg/L**^*a*^**	57 (21; 377)	
iAs, %**^*b*^**	8.7 ± 3.06	
MMA, %**^*b*^**	9.9 ± 3.4	
DMA, %**^*b*^**	81.3 ± 5.2	
**Maternal characteristics**		
Age at recruitment, years	26.6 ± 5.8	25.6 ± 5.3
BMI at GW8, kg/m^2^	20.4 ± 2.9	20.3 ± 2.9
Maternal education, years at school, n (%)		
No education	164 (25)	29 (22)
<5 years	71 (11)	14 (10)
≥5 - <10 years	305 (47)	62 (46)
≥ 10 years	100 (15)	29 (22)
SES quintiles, n (%)		
1 poorest	90 (14)	19 (15)
2	112 (18)	17 (12)
3	141 (22)	26 (19)
4	160 (25)	40 (30)
5 richest	137 (21)	32 (24)
Parity	1.34 ± 1.2	1.14 ± 1.2
Primiparous; n (%)	209 (33)	50 (37)
Multiparous; n (%)	431 (67)	84 (63)
Tobacco chewing during pregnancy, Yes/No; n (%)	49 (7)/591(93)	8 (7)/126(93)
U-As GW8, µg/L**^*a*^**	78 (20; 640)	78 (20; 447)
iAs, %**^*b*^**	14.9 ± 8.6	n.a.
MMA, %**^*b*^**	9.9 ± 4.9	n.a.
DMA, %**^*b*^**	75.1 ± 10.5	n.a.
U-As GW30, µg/L**^*a*^**	n.a.	71 (20; 535)
**Newborns**		
Gestational age at birth weeks	39.3 ± 1.9	39.3 ± 2.5
Birth weight, g	2746 ± 404	2820 ± 411
Low birth weight (<2500 g), n (%)	157 (25)	25 (19)
Birth length, cm	47.6 ± 2.1	48.1 ± 2.1
Boys/girls, n (%)	328 (51)/312(49)	71 (53)/63(47)

BMI, Body Mass Index; GW, Gestational week, HAZ, height-for-age z-score; WAZ, weight-for-age z-score; U-As; urinary arsenic. Data given as mean ± standard deviation, median (5; 95 percentiles), or n (%). ^a^Adjusted to average specific gravity of 1.012 g/mL; ^b^Percent of total metabolite concentration in urine.

### Plasma Biomarkers

Descriptive statistics of all analyzed plasma biomarkers are presented in Table 2. In spearman correlation plasma IGF-1 levels were positively associated with plasma Ca (r=0.171, P=0.06) and vitamin D levels (r=0.181, P=0.04) in newborns, but not at 4.5 yrs of age (r=0.002, P=0.96; r=0.013, P=0.743 respectively). Plasma PO_4_ concentration in 4.5 years old children correlated positively with PTH levels (r=0.109, P=0.006) and inversely with B-ALP (r=-0.187, P=0.0001). There was a positive association between plasma B-ALP and IGF-1 levels (r=0.111, P=0.005) in children. Girls had significantly higher IGF-1 and B-ALP levels in plasma compared to boys (Table 2). No significant difference in cord plasma biomarkers was observed in newborn boys and girls.

**Table 2:** Descriptive statistics of plasma growth biomarkers in children at 4.5 years of age and in cord blood, stratified by sex.

**Variables**	**4.5 years (N=640)**		**Cord blood (N=134)**	
	Boys (N=328)	Girls (N=312)	Boys (N=71)	Girls (N=63)
IGF, µg/L				
Mean ± SD	64 ± 41	72 ±37*	33 ± 16	38 ± 23
Median (5; 95 per)	53 (19; 135)	64 (23; 147)	31 (13; 59)	36 (8.1; 73)
Adj Ca, mg/dL				
Mean	7.7 ± 2.7	7.7 ± 3.1	5.3 ± 3.2	4.3 ± 2.8
Median	7.1 (4.6; 13.4)	7.1 (4.6; 13.2)	4.6 (1.1; 12.0)	3.3 (0.71; 10.2)
Vitamin D, nmol/L				
Mean	71 ± 24	70 ± 22	64 ± 29	68 ± 26
Median	68 (40; 118)	67 (41; 111)	59 (28; 133)	60 (36; 123)
PTH, ng/L				
Mean	44 ± 24	44 ± 20	5.0 ± 10.3	3.7 ± 5.4
Median	40 (20; 78)	40 (20; 80)	1.2 (1.1; 29)	1.2 (0.46; 20)
B-ALP, ug/L				
Mean	104 ± 50	114 ± 55*	17 ± 5.9	18 ± 8.9
Median	91 (47; 195)	101 (49; 217)	15 (10.4; 27)	16 (9.1; 41)
PO_4_, mg/dL				
Mean	21 ± 8	21 ± 7	25 ± 19	25 ± 16
Median	20 (12; 37)	20(13; 33)	17 (11; 79)	18 (12; 59)

IGF-1, Insulin-like growth factor 1; Adj Ca, albumin adjusted calcium; Vit-D, Vitamin D; PTH, Parathyroid hormone; B-ALP, Bone-specific alkaline phosphatase; PO_4_, phosphate. * indicates P<0.05 (Mann-Whitney U test) in comparing parameters between boys and girls.

### Arsenic exposure, child growth and plasma biomarkers

**Figure 1:**
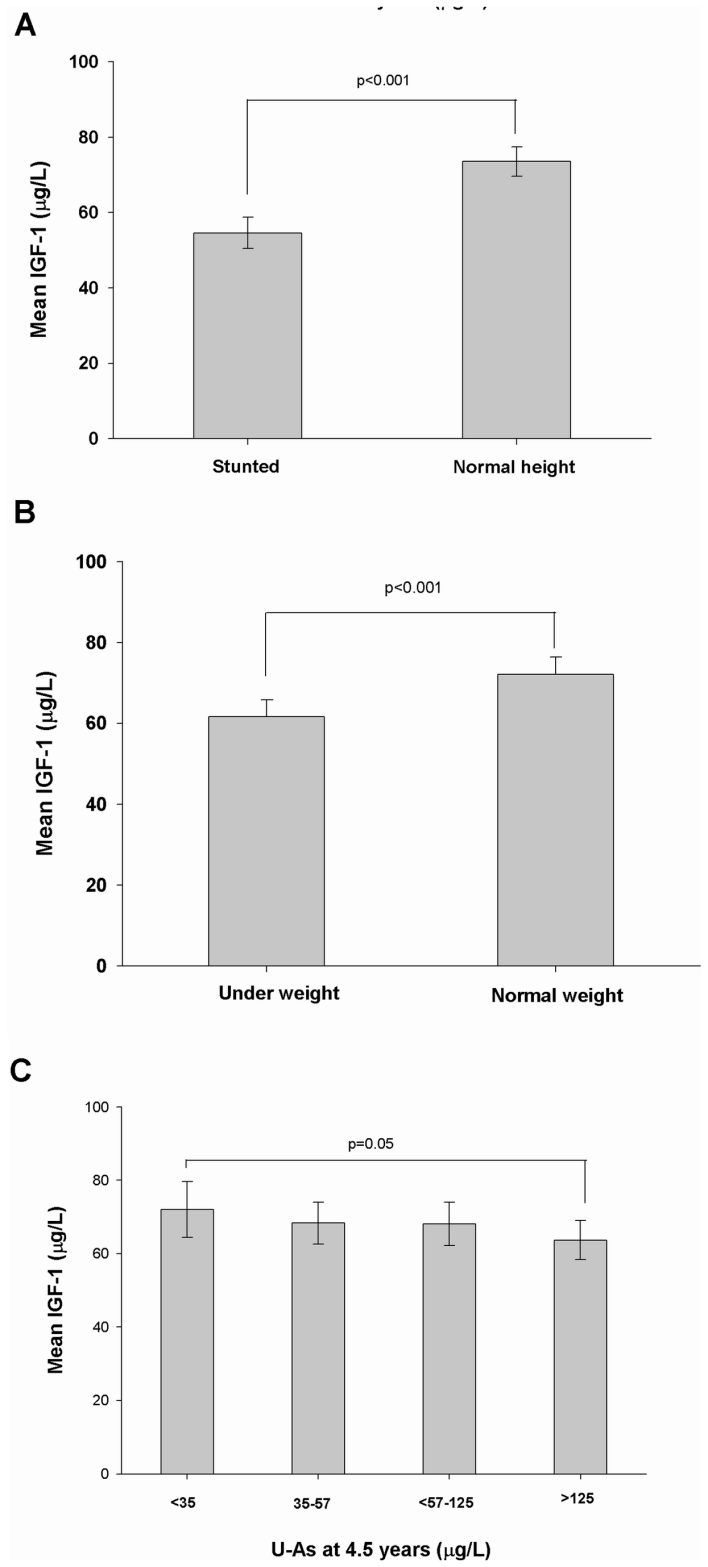
Mean insulin-like growth factor 1 (IGF-1) levels in (A) stunted (N=185) and normal height (N=455); (B) underweight (N=252) and normal weight (N=388) (; and (C) in different quartiles of urinary arsenic (N= 160 in each quartiles) in children at 4.5 years of age.

Child plasma IGF-1 levels correlated significantly with child height (r_s_=0.24; p<0.001) and weight (r_s_=0.17; p<0.001). Further evaluation of the basic characteristics of the children in relation to IGF-1 (median split at 59 μg/L) showed significantly lower height, weight, HAZ and WAZ (i.e. higher percentage of stunted and underweight children) with lower IGF-1 levels (Table 3). Such differences were, however, not found in the neonates. Both boys and girls with IGF-1 levels below 59 μg/L had significantly lower height, weight and HAZ, but not WAZ compared to those above the median split (Table S1 in File S1). Mean IGF-1 levels were significantly lower in stunted (Figure 1A) and underweight (Figure 1B) children compared to children with normal height and weight. No associations were obtained between growth indicators and other plasma biomarkers.

**Table 3:** Basic characteristics of 4.5 years old children and newborns in relation to plasma IGF-1 (median split at 59 µg/L).

**^*c*^Children 4.5 years (N=640)**	**IGF ≤59, µg/L (N=321)**	**IGF >59, µg/L (N=319)**	**P value (Mann- Whitney U)**
Height, Cm	99 ± 3.9	101 ± 3.9	<0.001
Weight, Kg	13.6 ± 1.4	14.1 ±1.7	<0.001
HAZ	-1.78 (-3.15; -0.35)	-1.44 (-2.73; 0.002)	<0.001
WAZ	-1.92 (-3.19; -0.56)	-1.62 (-2.96; -0.25)	<0.001
Stunted, n (%)	125 (39.4)	60 (18.8)	<0.001
Underweight, n (%)	142 (44.3)	110 (34.3)	0.012
U-As, µg/L**^*a*^**	59 (21; 411)	56 (21; 324)	0.20
iAs, %**^*b*^**	8.7 ± 3.1	8.8 ± 3.1	0.33
DMA, % **^*b*^**	81.6 ± 5.1	80.9 ± 5.4	0.09
MMA, % **^*b*^**	9.6 ± 3.4	10.2 ± 3.5	0.04
**^*d*^Newborns (N=134)**	**IGF ≤32, µg/L (N=68)**	**IGF >32, µg/L (N=66)**	
BW, g	2789 ± 427	2852 ± 395	0.30
BL, cm	47.8 ± 2.3	48.3 ± 1.9	0.11
HC, cm	31.9 ± 1.5	32.1 ± 1.5	0.51
CC, cm	31.3 ± 1.8	31.4 ± 2.1	0.46
U-As at GW8, µg/L**^*a*^**	85 (24; 443)	72 (17; 512)	0.13
iAs, % **^*b*^**	14.9 ± 5.3	13.7 ± 7.9	0.07
DMA, % **^*b*^**	75.0 ± 8.0	76.2 ± 9.2	0.33
MMA, % **^*b*^**	10.0 ± 4.1	10.1 ± 3.9	0.77
U-As at GW30, µg/L**^*a*^**	108 (21; 625)	63 (16; 562)	0.04

HAZ, height-for-age z-score; WAZ, weight-for-age z-score; U-As; urinary arsenic; BW, birth weight; BL, birth length; HC, head circumference; CC, chest circumference. Data given as mean ± standard deviation, median (5; 95 percentiles), or n (%). ^a^Adjusted to average specific gravity of 1.012 g/mL. ^b^Percent of total metabolite concentration in urine. ^c^Children at 4.5 years of age in relation to children blood IGF-1. ^d^Newborn characteristic in relation to cord blood IGF-1.

 When IGF-1 levels were compared between the lowest (<35 ug/L) v.s. the highest quartile (>125.5 ug/L) of U-As in 4.5 year old children, a significant reduction of IGF-1 levels were noted in the 4^th^ quartile compared to the 1^st^ quartile (P=0.05) (Figure 1C). There was a tendency of negative association between U-As and height (r=-0.067, P=0.08) and weight (r=-0.051, P=0.08) of children at 4.5 years of age. 

**Figure 2:**
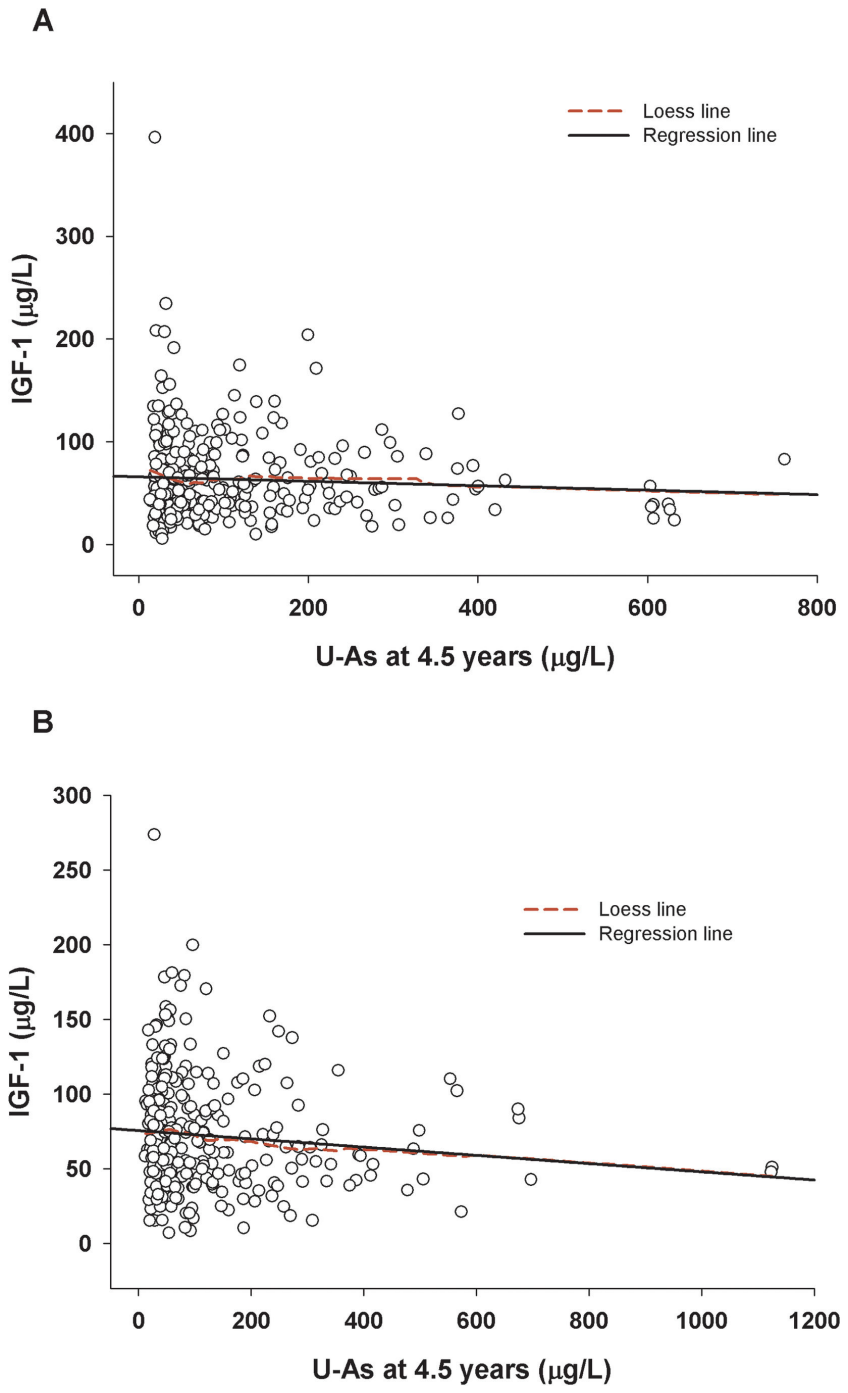
Associations between concurrent urinary arsenic (U-As) and plasma IGF-1 in 4.5 years children; (A) boys (r_s_=-0.06; P=0.26), (B) girls (r_s_=-0.118; P=0.038). Solid line indicates linear regression line and dashed line indicates Loess line.

In the linear regression analyses, concurrent U-As in children, but not prenatal arsenic, was inversely associated with child plasma IGF-1 at 4.5 years of age (Table 4; Figure 2A & B). The association remained fairly stable after adjustment for socioeconomic status (SES), parity (birth order), child sex, HAZ, and plasma levels of adjusted calcium (Adj Ca), Vit-D, PTH, B-ALP and PO_4_. None of the other plasma biomarkers showed significant associations with child U-As or maternal U-As during pregnancy. When stratified by sex, the association between child U-As and plasma IGF-1 was significant in girls only (Table 4). Plasma PO_4_ concentrations were significantly positively associated with U-As in boys.

**Table 4:** Linear regression analysis of plasma biomarkers in relation to concurrent and prenatal arsenic exposure in all children, boys and girls at 4.5 years age.

**Variables**	**U-As at 4.5 years (per 10 µg/L)**				**U-As at GW8 (per 10µg/L)**	
	Unadjusted		Adjusted		Adjusted	
	β (95% CI)	P	β (95% CI)	P	β (95% CI)	P
All children (N=640)						
IGF-1, µg/L^a^	- 0.24 (-0.48, -0.006)	0.044	-0.24 (-0.46, -0.02)	0.03	-0.004 (-0.11, 0.11)	0.94
IGF-1, µg/L^b^	- 0.24 (-0.46, -0.006)	0.044	-0.27 (-0.50, -0.042)	0.02	-0.008 (-0.12, 0.10)	0.89
Adj Ca, mg/dL^a^	0.009 (-0.007, 0.027)	0.25	0.007 (-0.009, 0.025)	0.36	0.004 (-0.005, 0.012)	0.42
Vit-D, nmol/L^a^	0.098 (-0.029, 0.22)	0.13	0.081 (-0.049, 0.201)	0.22	0.0023 (-0.068, 0.072)	0.94
PTH, ng/L^a^	-0.01 (-0.13, 0.11)	0.86	-0.016 (-0.13, 0.109)	0.81	0.028 (-0.038, 0.094)	0.40
B-ALP, µg/L^a^	0.098 (-0.19, 0.38)	0.50	0.084 (-0.21, 0.37)	0.57	-0.006 (-0.16, 0.15)	0.94
PO_4_, mg/dL^a^	0.023 (-0.02, 0.06)	0.30	0.020 (-0.023, 0.064)	0.36	0.013 (-0.011, 0.037)	0.27
Boys (N=328)						
IGF-1, µg/L^a^	-0.21 (-0.58, 0.15)	0.25	-0.23 (-0.57, 0.12)	0.20	-0.031 (-0.21, 0.15)	0.74
IGF-1, µg/L^b^	-0.21 (-0.58, 0.15)	0.25	-0.26 (-0.61, 0.10)	0.15	-0.041 (-0.22, 0.14)	0.65
Adj Ca, mg/dL^a^	-0.007 (-0.017¸ 0.032)	0.56	0.007 (-0.018, 0.032)	0.58	-0.0026 (-0.015, 0.009)	0.67
Vit-D, nmol/L^a^	0.031 (-.018, 0.25)	0.77	0.018 (-0.20, 0.23)	0.87	0.047 (-0.065, 0.15)	0.41
PTH, ng/L^a^	0.076 (-0.13, 0.29)	0.48	0.067 (-0.14, 0.28)	0.54	0.052 (-0.052, 0.15)	0.32
B-ALP, µg/L^a^	0.20 (-0.25, 0.65)	0.37	0.20 (-0.25, 0.65)	0.38	0.14 (-0.08, 0.37)	0.21
PO^4^, mg/dL^a^	0.088 (-0.010, 0.16)	0.02	0.085 (0.007, 0.16)	0.03	0.031 (-0.008, 0.071)	0.12
Girls (N=312)						
IGF-1, µg/L^a^	-0.27 (-0.56, 0.01)	0.05	-0.27 (-0.54, 0.011)	0.06	0.010 (-0.13, 0.15)	0.89
IGF-1, µg/L^b^	-0.27 (-0.56, 0.01)	0.05	-0.29 (-0.59, 0.021)	0.06	-0.023 (-0.12, 0.17)	0.76
Adj Ca, mg/dL^a^	0.011 (-0.011, 0.035)	0.03	0.005 (-0.018, 0.029)	0.66	0.0067 (-0.006, 0.020)	0.29
Vit-D, nmol/L^a^	-0.14 (-0.01, 0.29)	0.06	0.11 (-0.04, 0.26)	0.16	-0.040 (-0.13, 0.049)	0.38
PTH, ng/L^a^	0.06 (-0.20, 0.08)	0.39	-0.063 (-0.20, 0.082)	0.39	0.007 (-0.07, 0.09)	0.86
B-ALP, µg/L^a^	-0.005 (-0.37, 0.38)	0.98	0.026 (-0.36, 0.41)	0.89	-0.12 (-0.34, 0.10)	0.28
PO_4_, mg/dL**^*a*^**	-0.016 (-0.06, 0.032)	0.51	-0.022 (-0.072, 0.026)	0.36	-0.004 (-0.03, 0.024)	0.80

CI, confidence interval; β, unstandardized regression coefficients; U-As; urinary arsenic ; IGF-1, insulin-like growth factor 1; Adj Ca, albumin adjusted calcium; Vit-D, vitamin D; PTH, parathyroid hormone; B-ALP, bone-specific alkaline phosphatase; PO_4_, phosphate. ^a^Adjusted for SES, parity (birth order), child sex (for all children) and HAZ. ^b^Adjusted for SES, parity (birth order), child sex (for all children), HAZ, and plasma levels of Adj Ca, Vit-D, PTH, B-ALP and PO_4._

In the adjusted linear regression analyses stratified by stunting or underweight, U-As (per 10 µg/L) at 4.5 years was significantly inversely associated with plasma IGF-1 levels among children with normal height (β=-0.39;CI: -0.72, -0.07) or normal weight (β=-0.34; CI: -0.60, 0.005), but not in the stunted or underweight children (Table 5). When children with normal height were further stratified by sex, a significant inverse association between U-As and IGF-1 and a positive association between U-As and plasma Ca levels were found in girls only (Table 5). Similarly, when children with normal weight were stratified by sex, a significant inverse association was found between U-As and IGF-1 in only girls. In accordance with this, when stratified by SES, a similar association was found in children with high SES, particularly in girls.

**Table 5:** Linear regression analysis of associations between concurrent arsenic exposure and plasma growth biomarkers in 4.5 yr old children in relation to growth retardation and socioeconomic status.

	**U-As at 4.5 years (per 10 µg/L)**					
	Stunted (N=184)	Adequate height (N=449)	Underweight (N=247)	Adequate weight (N=386)	Low SES (N=323)	High SES (N=312)
	β (95 % CI)	β (95 % CI)	β (95 % CI)	β (95 % CI)	β (95 % CI)	β (95 % CI)
All children (N=640)						
IGF-1 (µg/L)**^*a*^**	-0.016 (-0.2, 0.23)	-0.37 (-0.6, -0.05)*	-0.15 (-0.4, 0.11)	-0.30 (-0.6¸ 0.04)	-0.11 (-0.3, 0.13)	-0.46 (-0.89, -0.02)*
IGF-1 (µg/L)**^*b*^**	0.009 (-0.28, 0.3)	-0.39 (-0.72, 0.07)	-0.14 (-0.4, 0.17)	-0.34 (-0.6¸ 0.005)	-0.10 (-0.36, 0.16)	-0.51 (-0.94, -0.07)*
Adj Ca (mg/dL) **^*a*^**	-0.005 (-0.04¸0.03)	0.013 (-0.006, 0.03)	0.003 (-0.02, 0.03)	0.012 (-0.01, 0.03)	0.004 (-0.02, 0.02)	0.015 (-0.01¸ 0.04)
PO_4_ (mg/dL) **^*a*^**	0.054 (-0.01, 0.12)	0.002 (-0.05, 0.059)	0.092 (0.03, 0.14)*	-0.034 (-0.09¸ 0.03)	0.045 (-0.01¸ 0.1)	-0.035 (-0.10, 0.03)
Boys (N=328)						
IGF-1 (µg/L) **^*a*^**	0.047 (-0.41¸ 0.51)	-0.29 (-0.73, 0.15)	-0.18 (-0.69, 0.31)	-0.21 (-0.70,0 .27)	0.035 (-0.3, 0.38)	-0.36 (-1.03, 0.31)
IGF-1 (µg/L) **^*b*^**	0.011 (-0.53, 0.55)	-0.32 (-0.77¸0 .13)	-0.12 (-0.70¸ 0.45)	-0.23 (-0.73, 0.25)	0.028 (-0.33¸0.4)	-0.47 (-1.16¸0.21)
Adj Ca (mg/dL) **^*a*^**	0.059 (-0.001, 0.1)	-0.005 (-0.03¸ 0.02)	0.026 (-0.03, 0.08)	0.002 (-0.02, 0.03)	0.013 (-0.02, 0.04)	0.0014 (-0.040, 0.04)
PO_4_ (mg/dL) **^*a*^**	0.27 (0.089, 0.46)*	0.045 (-0.041, 0.13)	0.31 (0.21¸ 0.41)*	-0.008 (-0.11, 0.09)	0.18 (0.06¸ 0.30)*	-0.035 (-0.13¸ 0.062)
Girls (N=312)						
IGF-1 (µg/L) **^*a*^**	0.035 (-0.35, 0.28)	-0.51 (-0.9, -0.04)*	-0.16 (-0.50¸0.17)	-0.44 (-0.92, 0.03)	-0.19 (-0.54, 0.15)	-0.56 (-1.09, -0.03)*
IGF-1 (µg/L) **^*b*^**	0.0054 (-0.39, 0.4)	-0.49 (-0.97¸-0.02)*	-0.13 (-0.54¸0 .27)	-0.47 (0.97¸ 0.01)*	-0.18 (-0.58, 0.21)	-0.55 (-1.1¸ -0.004)*
Adj Ca (mg/dL) **^*a*^**	-0.03 (-0.07, 0.02)	0.026 (.002¸ .051)*	-0.008 (-0.04, 0.03)	0.016 (-0.01, 0.04)	-0.0062 (-0.03¸ 0.02)	0.033 (-0.007¸ 0.07)
PO_4_ (mg/dL) **^*a*^**	0.005 (-0.06¸0.069)	-0.045 (-0.11, 0.02)	0.013 (-0.04¸ .007)	-0.06 (-0.13¸ 0.014)	-0.017 (-0.08, 0.04)	-0.034 (-0.12¸ 0.055)

CI, confidence interval; β, unstandardized regression coefficients; U-As, urinary arsenic; SES, socioeconomic status; IGF-1, insulin-like growth factor 1; Adj Ca, albumin adjusted calcium; Vit-D, vitamin D; PTH, parathyroid hormone; B-ALP, bone-specific alkaline phosphatase; PO_4_, phosphate. **^*a*^**Adjusted for SES, parity (birth order), child sex (for all children). **^*b*^**Adjusted for SES, parity (birth order), child sex (for all children), and plasma levels of Adj Ca, Vit-D, PTH, B-ALP and PO_4_. * indicates P<0.05.

To evaluate the combined effects of prenatal and concurrent exposure, we entered both exposures in the same model (although both exposures were correlated) with other adjusting covariates, and found that only concurrent (but not prenatal) exposure was significant in the model. In this case, the estimate for concurrent exposure increased about 8% (data not shown).

The analyses stratified by sex showed stronger positive associations between child U-As and plasma PO_4_ levels in stunted or underweight boys compared to those with normal height or weight (Table 5). These findings were further supported in the analyses stratified by SES where PO_4_ levels in boys with lower SES were positively associated with child U-As (Table 5). A positive association between U-As and plasma Ca levels were observed in stunted boys only.

We also evaluated the impact of arsenic methylation efficiency (pattern of urinary arsenic metabolites) on the association between U-As and IGF-1. Since we found a significantly higher concentration of %MMA (U-As metabolite) in the high IGF-1 group (median split at 59 μg/L) compared to the low IGF-1 group (Table 3), we additionally adjusted for % of MMA. The estimates of the associations between U-As and IGF-1 changed about 15% (Table S2 in File S1). Thereafter, we stratified the analysis by %MMA (median split at 9.7%) and an inverse association between U-As and IGF-1 was found in all children with high %MMA. When further stratified by sex and growth, the association was robust in girls (Table 6) particularly those with normal height (Table S3 in File S1). Boys showed no major impact of %MMA in urine, besides a slightly clearer association between U-As and plasma PO_4_ in the high %MMA group (Table 6).

**Table 6:** Linear regression analysis of plasma biomarkers in relation to concurrent arsenic exposure in 4.5 years old children, boys and girls stratified by % of MMA (median split, 9.7%).

**Variables**	**U-As at 4.5 years (per 10 µg/L)**			
	Low % of MMA		High % of MMA	
	β (95% CI)	P	β (95% CI)	P
All children (N=640)				
IGF-1, µg/L**^*a*^**	-0.12 (-0.48, 0.23)	0.58	-0.34(-0.62, -0.05)	0.02
IGF-1, µg/L**^*b*^**	-0.15 (-0.56, 0.27)	0.48	-0.36(-0.64, -0.07)	0.01
Adj Ca, mg/dL**^*a*^**	0.026 (-0.009, 0.06)	0.15	0.0006(-0.018, 0.019)	0.95
Vit-D, nmol/L**^*a*^**	0.008 (-0.20, 0.21)	0.94	0.06 (-0.10, 0.22)	0.47
PTH, ng/L**^*a*^**	0.013 (-0.21, 0.23)	0.90	-0.033 (-0.17, 0.11)	0.65
B-ALP, µg/L**^*a*^**	-0.10 (-0.62, 0.41)	0.69	0.17 (-0.18, 0.52)	0.35
PO_4_, mg/dL**^*a*^**	0.019 (-0.06, 0.10)	0.64	0.028 (-0.02, 0.07)	0.26
Boys (N=328)				
IGF-1, µg/L**^*a*^**	-0.21 (-0.77, 0.35)	0.46	-0.25 (-0.70, 0.20)	0.28
IGF-1, µg/L**^*b*^**	-0.23 (-0.79, 0.34)	0.43	-0.37 (-0.84, 0.096)	0.11
Adj Ca, mg/dL**^*a*^**	0.028 (-0.01, 0.065)	0.15	-0.009 (-0.04, 0.02)	0.60
Vit-D, nmol/L**^*a*^**	-0.032 (-0.32, 0.26)	0.82	0.012 (-0.31, 0.34)	0.94
PTH, ng/L**^*a*^**	0.047 (-0.31, 0.40)	0.79	0.088 (-0.16, 0.34)	0.49
B-ALP, µg/L**^*a*^**	0.007 (-0.71, 0.73)	0.98	0.41 (-0.17, 0.99)	0.16
PO_4_, mg/dL**^*a*^**	0.086 (-0.049, 0.22)	0.21	0.089 (.0.003, .017)	0.04
Girls (N=312)				
IGF-1, µg/L**^*a*^**	-0.042 (-0.51, 0.41)	0.85	-0.45 (-0.81, -0.08)	0.01
IGF-1, µg/L**^*b*^**	-0.017 (-0.67, 0.64)	0.95	-0.41 (-0.77, -0.036)	0.03
Adj Ca, mg/dL**^*a*^**	0.019 (-0.04, 0.08)	0.55	0.0034 (-0.019, 0.026)	0.76
Vit-D, nmol/L**^*a*^**	0.049 (-0.25, 0.35)	0.75	0.079 (-0.096, 0.25)	0.37
PTH, ng/L**^*a*^**	-0.025 (-0.29, 0.24)	0.85	-0.08 (-0.26, 0.10)	0.38
B-ALP, µg/L**^*a*^**	-0.19 (-0.95, 0.56)	0.61	0.076 (-0.38, 0.54)	0.74
PO_4_, mg/dL**^*a*^**	-0.046 (-0.13, 0.043)	0.31	-0.005 (-.0.064, 0.05)	0.85

CI, confidence interval; β, unstandardized regression coefficients; U-As; urinary arsenic ; IGF-1, insulin-like growth factor 1; Adj Ca, albumin adjusted calcium; Vit-D, vitamin D; PTH, parathyroid hormone; B-ALP, bone-specific alkaline phosphatase; PO_4_, phosphate; MMA, monomethyl arsonic acid; SES, socioeconomic status. ^a^Adjusted for SES, parity (birth order), child sex (for all children) and HAZ. ^b^Adjusted for SES, parity (birth order), child sex (for all children), HAZ, and plasma levels of Adj Ca, Vit-D, PTH, B-ALP and PO_4_.

### As exposure and IGF-1 in neonates

**Figure 3:**
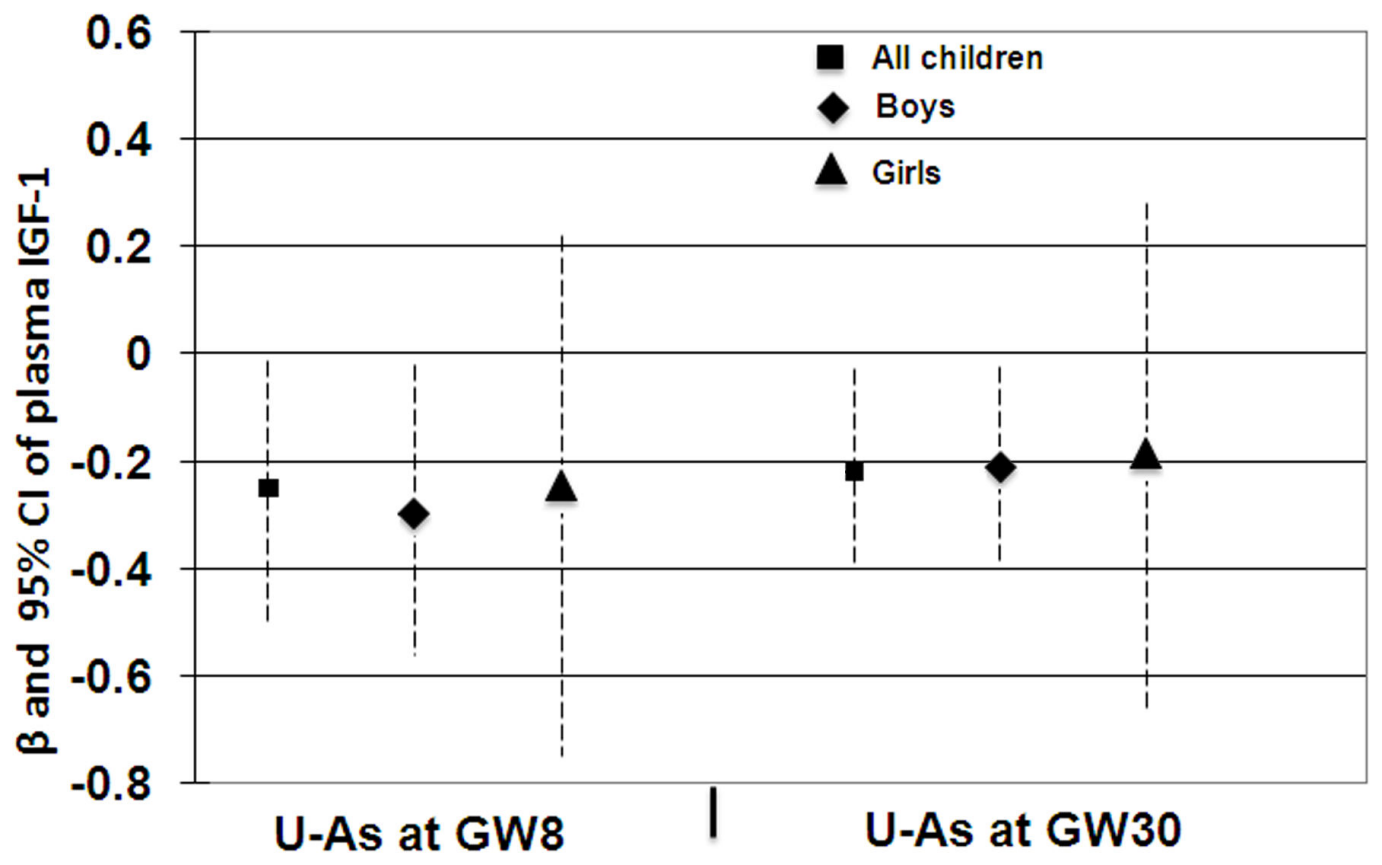
Associations between maternal urinary arsenic at gestational week (GW) 8 and 30 with cord blood insulin-like growth factor 1 (IGF-1) in all children, boys and girls. The associations were adjusted with mother age, socioeconomic status (SES), parity (birth order), child sex (for all children), and birth weight and height.

Prenatal As exposure (maternal U-As at GW8 and GW30) was inversely associated with plasma concentrations of IGF-1 in neonates at birth (r=-0.242, P=0.005 and r=-0.254, P=0.003 respectively). Multi-variable adjusted linear regression analysis of maternal U-As at GW8 or GW30 showed a significantly inverse association with IGF-1 levels in neonates (Figure 3). Intriguingly, the significant association was evident in boys only when stratified by sex. 

### Nutritional supplementation groups

 We evaluated the effects of concurrent arsenic exposure on plasma biomarkers at 4.5 years of age in different maternal supplementation groups. There was a tendency of lower plasma IGF-1 in relation to concurrent arsenic exposure in the 60 mg Fe supplementation group (β= -0.543, P= 0.035). We did not find any significant associations between concurrent arsenic exposure and other plasma biomarkers in different supplementation groups.

## Discussion

In this study, we found that arsenic exposure was associated with lower plasma IGF-1 in pre-school children. The effect was more evident in girls, particularly adequately nourished girls, than in boys. This finding is essential for our understanding of the mode of action of the previously-reported impairments in fetal and child growth associated with moderately elevated arsenic exposure [6-8,15,16]. 

The suppressive effects on child growth in the same but larger cohort were stronger for concurrent exposure than for prenatal exposure, and consistently affected growth in girls more than in boys, particularly more in girls with higher SES [8,15]. We also found similar arsenic related effects on child IGF-1 in the present study. *In utero* arsenic exposure, on the other hand, was associated with lower cord blood IGF-1 concentrations in boys (Figure 3), in accordance with our previous reports on arsenic exposure and lower fetal size in boys [6]. On average, boys had 12% lower IGF-1 than girls, both at birth and at 4.5 years of age, which is in line with previous findings [17,18] and is also supported by an earlier study showing gender specificity of IGF-1 levels at term birth [19]. Similarly, among healthy Turkish and Korean children of the same age group as ours, IGF-1 levels in girls (162 and 160 μg/L, respectively) were generally higher than in boys (115 and 145 ug/L, respectively) [20,21]. It is particularly noteworthy that IGF-1 levels of girls (72± 37 μg/L) and boys (64± 41 μg/L) in our cohort were almost half of that of healthy children of the same age group from resource rich countries such as Turkey and Korea. Girls in general are more growth retarded than boys, especially in developing countries. Similarly, girls in our cohort are more stunted (33% vs 26%) and more underweight (43% vs 36%) than boys. It may be assumed that in order to compensate for the short stature of the girls, IGF-1 levels may remain elevated during the rapidly growing years of childhood. Other factors that are known to affect the levels of IGF-1 include genetics, stress, nutrition, body mass index, and disease status, including xenobiotic intake [22]. To the best of our knowledge, this is the first indication of adverse effects of arsenic on child IGF-1. The finding is supported by *in vitro* studies with differentiating C2C12 cells, which after exposure to 20 nM arsenic, showed epigenetic effects of altered histone remodeling status on the myogenin promoter and impaired protein and mRNA levels of IGF-1[23]. 

 IGF-1 is a primary mediator of the effects of growth hormone and plays an important role in childhood growth. As expected, we found a strong positive association between plasma IGF-1 levels and height and weight. The majority of stunted and underweight children in this study cohort were present in the low IGF-1 group compared to the high IGF-1 group (stunted, 39% vs 19%, P<0.001; underweight, 44% vs 34%, P=0.012 respectively). In an earlier study, Fall et al showed higher plasma concentrations of IGF-1 in children who were underweight *in utero*[18]. We did not find any differences in the IGF-1 levels between low birth weight and normal weight children at birth or at 4.5 yrs of age. It is quite likely that arsenic may re-program the effects of IGF-1 that consequently affects fetal growth. However, further studies are needed to confirm this postulation. Nutrition is an important determinant for child growth; this may explain our finding of stronger associations with elevated arsenic exposure in the girls with better nutrition (normal height and weight) than in those with poor nutrition. It is possible that in stunted and growth-retarded children, the negative effects of As exposure on IGF-1 are masked by multiple factors contributing to growth retardation, such as recurrent infections, malnutrition, micronutrient deficiencies, poverty, and stress, whereas in children with normal growth, the suppressive effects are more obvious. It was also not clear why %MMA was higher in the high IGF group compared to the low IGF group. One may assume that the toxic effects of higher MMA levels in children may be compensated with higher production of IGF-1.

 Recent studies indicate that cord blood IGF-1 is a key regulator of neonatal immune responses in maturation and infection, particularly by suppressing pro-inflammatory Th1 responses [24]. Interestingly, we previously found that prenatal arsenic exposure was associated with an increased incidence of acute respiratory infections in male infants [25], and with cold and cough later in childhood [8], possibly related to an impaired thymus function [26]. Disregulation or disturbances of IGF-1 levels in serum and its receptor may be linked with susceptibility to infections and subsequent diseases in children.

 IGF-1 signaling is important for linear and appositional bone growth, bone mineralization [27] and bone accrual during the postnatal growth phase [28]. An association between a reduced plasma concentration of IGF-1 and the inhibition of bone and muscle growth has been demonstrated in studies in animals [29]. Bone tissue is also a target organ for arsenic since it is an analog of PO_4_ [14,30,31]. Arsenic and phosphorus are located in the same group of the periodic table, and arsenic can compete with phosphorus in the oxidative phosphorylation process that can lead to replacement of phosphorus in the bone [9,32,33]. Indeed, we found elevated plasma PO_4_ levels to be related to arsenic exposure in boys, particularly malnourished boys. The potential consequences of this in terms of bone health remain to be investigated. 

 One limitation of this study is the small sample size of the neonatal cohort, resulting in low statistical power, especially after stratifying the data by sex. In this study, there was a trend for an inverse association between current arsenic exposure and growth indicators; however, the association was not significant. Probably, a much larger sample size is needed to find a significant association between arsenic exposure and anthropometric indicators as seen for children at birth and 2 years of age in the MINIMat cohort [8,15]. We did not find any association between growth indices and plasma biomarkers other than IGF-1.

 In conclusion, our study showed that prenatal arsenic exposure has suppressive effects on cord blood IGF-1 in boys at birth, while childhood exposure impaired IGF-1 at 4.5 years of age, particularly in girls, at an age when more girls were stunted and underweight than boys. These findings may explain, in part, the previously-reported gender-specific impairment of arsenic exposure on fetal and child growth. Further studies on growing children at pre-adolescent stages are ongoing to better understand and decipher the role of arsenic in child growth trajectory.

## Materials and Methods

### Ethics Statement

 The study (Research Protocol # 2008-034) was approved by the ethical review committee (ERC) of icddr,b on 7^th^ August, 2008. Written informed consent was obtained from the participating women and the parents/ guardians on behalf of the children.

### Study area and subjects

 The study was conducted in Matlab, a rural riverine area located 53 km southeast of Dhaka, Bangladesh. The International Centre for Diarrhoeal Disease Research, Bangladesh (icddr,b) runs a central hospital and four sub-center clinics and maintains a Health and Demographic Surveillance System (HDSS) in this area since 1966. Matlab is extensively affected by arsenic contamination of drinking water. A population-based survey in 2002-2003 measuring arsenic concentrations in all functioning 13,286 tubewells in Matlab found about 70% of the tube-wells installed during the last few decades exceeding the WHO drinking water guidelines of 10 µg As/L [34,35]. 

 Our ongoing research on effects of early-life arsenic exposure [25,26,36] was nested in a large, randomized, population-based food and multi-micronutrient supplementation trial (MINIMat trial; ISRCTN 16581394), which evaluated nutritional impacts on pregnancy outcomes and child health [37]. We obtained maternal urine samples in early pregnancy (average gestational week, GW8), late pregnancy (average GW30, n=134) and cord blood at delivery. Field research assistants collected information on socioeconomic status (SES), parity, and tobacco use based on a set of structured questionnaires given to the pregnant women during the scheduled monthly home visits. 

 The present study included 640 children, born during 2003-2004, and followed-up at 4.5 years of age with both anthropometry and growth biomarkers. In a subset (N=134) of children we had data on growth markers at birth. This subset included pregnant women who delivered singleton infants at the central Matlab hospital or any of the four sub-center clinics during early in the day (5:00 AM to 2:30 PM). This design was due to the necessity to transport cord blood samples and process them in the laboratory within working hours of the same day. Birth weight was measured within 72 hours of delivery using electronic scales (SECA pediatric scales, U.K.) with precision of 10g. Infant length was measured using a validated, locally-manufactured wooden length board, with precision of 0.1 cm.

 Fasting blood was collected from 4.5 years old children in the sub-center clinics in trace element-free lithium-heparin tubes. Body weight, in light clothing and bare feet, was measured to the nearest 0.1 kg with a digital scale (TANITA HD -318, Tanita Corporation, Japan). Height was measured to the nearest 0.1 cm with the use of a stadiometer Leicester Height Measure (Seca 214, UK). Weight and height measurements were converted to weight-for-age, height-for-age, and weight-for-height Z-scores (SD scores). Stunting was defined as <-2 Z-score for height-for-age and underweight was defined as <-2 Z-score for weight-for-age [38] (http://www.who.int/childgrowth/software/en/). 

 Information of the SES of the family was collected using a wealth index that was based on information about household assets, house construction materials, ownership of land, source of income of the household and family characteristics such as parental education etc. The wealth index was estimated by principal component analysis, producing a weighted score [39]. Scores were categorized into quintiles, with category 1 representing the poorest and category 5 the richest. Due to limited power of the analysis, the SES scores were divided into two groups on the basis of the median split. 

### Arsenic exposure

 We measured concentrations of metabolites of inorganic As in maternal urine (U-As; defined as the sum of inorganic arsenic and its methylated forms, monomethyl arsonic acid (MMA) and dimethylarsinic acid (DMA)), that reflects the ingested dose of iAs from all sources [35]. The relative fractions of urinary metabolites were used as markers of arsenic methylation efficiency. Urinary As in early (GW8) pregnancy was used as a marker of fetal exposure [40]. Urine was collected into trace-element-free plastic cups, transferred to 24 mL polyethylene bottles, and stored at -70°C. The urinary arsenic concentrations in mothers and their children were measured using high-performance liquid chromatography online with hydride generation and inductively coupled plasma mass spectrometry, as described previously [41,42]. For quality control purposes, we analyzed NIES reference material (CRM No.18, National Institute for Environmental Studies, Tsukuba City, Japan) together with urine samples collected from mothers and 4.5 years old children. The certified reference value of DMA was 36 ± 9 µg/L (mean ± SD) and the DMA concentration we obtained was 43 ± 1.7 µg/L in maternal urine and 42 ± 1.2 µg/L in children. To compensate for variation in dilution, urine samples were adjusted to the average specific gravity (both for mothers and children, 1.012 g/mL), measured by a digital refractometer (EUROMEX RD712 Clinical Refractometer, Holland) [43].

### Biomarkers of growth

Plasma was separated from blood cells by centrifugation and plasma aliquot was stored at –80 °C. Plasma levels of IGF-1, 25-OH VD, B-ALP, and iPTH were measured using the Human IGF-1 ELISA kit (Quantakine ELISA, R&D Systems, Inc., Minneapolis, MN), 25-hydroxy vitamin D EIA kit (Immunodiagnostic Systems Ltd., Boldon, UK), Ostase BAP kit (Immunodiagnostic Systems Ltd., Boldon, UK), and the PTH Intact ELISA kit (DRG International Inc., USA), respectively, according to the manufacturer’s instructions. All absorbance were measured at 450 nm (reference 650 nm, wavelength correction set at 540) using a microplate reader. The concentrations were calculated based on the standard curves.

Ca in plasma was measured using the QuantiChrom calcium assay kit (Bioassay Systems, Hayward, CA) and adjusted for plasma albumin concentrations since about half of serum Ca is bound to albumin [44]. Plasma albumin concentration was determined by the ALB plus kit (Roche Diagnostics GmbH, Mannheim, Germany) in the automated clinical chemistry autoanalyzer (Hitachi 902, Hitachi Ltd, Tokyo, Japan). Bi-level control serum of normal level and high level, Precinorm Protein and Precipath Protein (Roche diagnostics GmbH) were used to check both accuracy and precision for albumin. For B-ALP, iPTH and Vitamin D, commercially available control serum from the respective kit manufacturer was used. Co-efficient of variation was 5.6% for IGF-1, 8.6% and 5.2% for Ca, 6.8% and 4.8% for BAP, 11.0% and 10.3% for PTH, 10.5% and 10.4% for vitamin D, and 6.6% and 1.6% for albumin.

### Statistical analysis

 Statistical analyses were performed using the software PASW 20.0 (SPSS Inc., Chicago, USA) and Stata/IC, version 12.0 (StataCorp, Texas, USA). Data distribution patterns were evaluated by scatter plots, and normality and homogeneity of variances were checked. Associations between arsenic exposure (per 10 µg/L) (urinary arsenic metabolites) and growth biomarkers were analyzed in multivariable-adjusted regression models, controlling for potential confounders and influencing factors. Confounders were identified from the covariates (maternal age, height, weight, body mass index (BMI), gestational age, parity/birth order, SES, tobacco chewing, as well as sex, birth weight and size, WHZ, HAZ and WAZ of the children) based on correlations (*p* < 0.2) with the exposure (As) and/ or outcome, and were included in final models when they changed the effect estimate for U-As on the outcome by 5% or more. Mothers’ age, parity, SES and sex of the baby, were included in the final models. Furthermore, IGF-1 was additionally adjusted by other plasma biomarkers. *P*-values < 0.05 were considered significant.

 In the sensitivity analysis, all plasma biomarkers were adjusted for other plasma biomarkers.

## Supporting Information

File S1 **Supporting files.**
(DOCX)

## References

[1] Ahmad SA, Sayed MH, Barua S, Khan MH, Faruquee MH et al. (2001) Arsenic in drinking water and pregnancy outcomes. Environ Health Perspect 109: 629-631. doi:10.1289/ehp.01109629. PubMed: 11445518. 11445518 PMC1240346

[2] Hopenhayn-Rich C, Browning SR, Hertz-Picciotto I, Ferreccio C, Peralta C et al. (2000) Chronic arsenic exposure and risk of infant mortality in two areas of Chile. Environ Health Perspect 108: 667-673. doi:10.1289/ehp.00108667. PubMed: 10903622. 10903622 PMC1638185

[3] Milton AH, Smith W, Rahman B, Hasan Z, Kulsum U et al. (2005) Chronic arsenic exposure and adverse pregnancy outcomes in Bangladesh. Epidemiology 16: 82-86. doi:10.1097/00001648-200509000-00205. PubMed: 15613949. 15613949

[4] Rahman A, Vahter M, Ekström EC, Rahman M, Golam Mustafa AH et al. (2007) Association of arsenic exposure during pregnancy with fetal loss and infant death: a cohort study in Bangladesh. Am J Epidemiol 165: 1389-1396. doi:10.1093/aje/kwm025. PubMed: 17351293. 17351293

[5] Yang CY, Chang CC, Tsai SS, Chuang HY, Ho CK et al. (2003) Arsenic in drinking water and adverse pregnancy outcome in an arseniasis-endemic area in northeastern Taiwan. Environ Res 91: 29-34. doi:10.1016/S0013-9351(02)00015-4. PubMed: 12550085. 12550085

[6] Kippler M, Wagatsuma Y, Rahman A, Nermell B, Persson LA et al. (2012) Environmental exposure to arsenic and cadmium during pregnancy and fetal size: a longitudinal study in rural Bangladesh. Reprod Toxicol 34: 504-511. doi:10.1016/j.reprotox.2012.08.002. PubMed: 22985739. 22985739

[7] Rahman A, Vahter M, Smith AH, Nermell B, Yunus M et al. (2009) Arsenic exposure during pregnancy and size at birth: a prospective cohort study in Bangladesh. Am J Epidemiol 169: 304-312. PubMed: 19037006. 19037006 10.1093/aje/kwn332

[8] Gardner RM, Kippler M, Tofail F, Bottai M, Hamadani J et al. (2013) Combined environmental exposures to metals and children’s growth to five years: a prospective cohort study. American J Epidemiol (In press). 10.1093/aje/kws437 PMC3676155 23676282

[9] Lindgren A, Vahter M, Dencker L (1982) Autoradiographic studies on the distribution of arsenic in mice and hamsters administered 74As-arsenite or -arsenate. Acta Pharmacol Toxicol (Copenh) 51: 253-265. 7136731 10.1111/j.1600-0773.1982.tb01023.x

[10] Lindgren A, Danielsson BR, Dencker L, Vahter M (1984) Embryotoxicity of arsenite and arsenate: distribution in pregnant mice and monkeys and effects on embryonic cells in vitro. Acta Pharmacol Toxicol (Copenh) 54: 311-320. PubMed: 6730986. 6730986 10.1111/j.1600-0773.1984.tb01936.x

[11] Hill DS, Wlodarczyk BJ, Finnell RH (2008) Reproductive consequences of oral arsenate exposure during pregnancy in a mouse model. Birth Defects Res B Dev Reprod Toxicol 83: 40-47. doi:10.1002/bdrb.20142. PubMed: 18186108. 18186108

[12] Willhite CC (1981) Arsenic-induced axial skeletal (dysraphic). Disorders - Exp Mol Pathol 34: 145-158. doi:10.1016/0014-4800(81)90071-X. 6894125

[13] Lammon CA, Hood RD (2004) Effects of protein deficient diets on the developmental toxicity of inorganic arsenic in mice. Birth Defects Res B Dev Reprod Toxicol 71: 124-134. doi:10.1002/bdrb.20006. PubMed: 15282733. 15282733

[14] Odstrcil Adel C, Carino SN, Ricci JC, Mandalunis PM (2010) Effect of arsenic in endochondral ossification of experimental animals. Exp Toxicol Pathol 62: 243-249. PubMed: 19447590. 19447590 10.1016/j.etp.2009.04.001

[15] Saha KK, Engström A, Hamadani JD, Tofail F, Rasmussen KM et al. (2012) Pre- and postnatal arsenic exposure and body size to 2 years of age: a cohort study in rural Bangladesh. Environ Health Perspect 120: 1208-1214. doi:10.1289/ehp.1003378. PubMed: 22504586. 22504586 PMC3440068

[16] Hopenhayn C, Ferreccio C, Browning SR, Huang B, Peralta C et al. (2003) Arsenic exposure from drinking water and birth weight. Epidemiology 14: 593-602. doi:10.1097/01.ede.0000072104.65240.69. PubMed: 14501275. 14501275

[17] Casazza K, Higgins PB, Fernández JR, Goran MI, Gower BA (2008) Longitudinal analysis of the insulin-like growth factor system in African-American and European American children and adolescents. J Clin Endocrinol Metab 93: 4917-4923. doi:10.1210/jc.2008-0999. PubMed: 18782874. 18782874 PMC2626444

[18] Fall CH, Pandit AN, Law CM, Yajnik CS, Clark PM et al. (1995) Size at birth and plasma insulin-like growth factor-1 concentrations. Arch Dis Child 73: 287-293. doi:10.1136/adc.73.4.287. PubMed: 7492190. 7492190 PMC1511321

[19] Ibáñez L, Sebastiani G, Lopez-Bermejo A, Díaz M, Gómez-Roig MD et al. (2008) Gender specificity of body adiposity and circulating adiponectin, visfatin, insulin, and insulin growth factor-I at term birth: relation to prenatal growth. J Clin Endocrinol Metab 93: 2774-2778. doi:10.1210/jc.2008-0526. PubMed: 18460569. 18460569

[20] Hyun SE, Lee BC, Suh BK, Chung SC, Ko CW et al. (2012) Reference values for serum levels of insulin-like growth factor-I and insulin-like growth factor binding protein-3 in Korean children and adolescents. Clin Biochem 45: 16-21. doi:10.1016/j.clinbiochem.2011.10.003. PubMed: 22032863. 22032863

[21] Yüksel B, Özbek MN, Mungan NO, Darendeliler F, Budan B et al. (2011) Serum IGF-1 and IGFBP-3 levels in healthy children between 0 and 6 years of age. J Clin Res Pediatr Endocrinol 3: 84-88. doi:10.4274/jcrpe.v3i2.17. PubMed: 21750637. 21750637 PMC3119446

[22] Scarth JP (2006) Modulation of the growth hormone-insulin-like growth factor (GH-IGF) axis by pharmaceutical, nutraceutical and environmental xenobiotics: an emerging role for xenobiotic-metabolizing enzymes and the transcription factors regulating their expression. A review. Xenobiotica 36: 119-218. doi:10.1080/00498250600621627. PubMed: 16702112. 16702112

[23] Hong GM, Bain LJ (2012) Sodium arsenite represses the expression of myogenin in C2C12 mouse myoblast cells through histone modifications and altered expression of Ezh2, Glp, and Igf-1. Toxicol Appl Pharmacol 260: 250-259. doi:10.1016/j.taap.2012.03.002. PubMed: 22426358. 22426358 PMC3335964

[24] Puzik A, Rupp J, Tröger B, Göpel W, Herting E et al. (2012) Insulin-like growth factor-I regulates the neonatal immune response in infection and maturation by suppression of IFN-gamma. Cytokine 60: 369-376. doi:10.1016/j.cyto.2012.07.025. PubMed: 22898392. 22898392

[25] Raqib R, Ahmed S, Sultana R, Wagatsuma Y, Mondal D et al. (2009) Effects of in utero arsenic exposure on child immunity and morbidity in rural Bangladesh. Toxicol Lett 185: 197-202. doi:10.1016/j.toxlet.2009.01.001. PubMed: 19167470. 19167470

[26] Ahmed S, Ahsan KB, Kippler M, Mily A, Wagatsuma Y et al. (2012) In utero arsenic exposure is associated with impaired thymic function in newborns possibly via oxidative stress and apoptosis. Toxicol Sci 129: 305-314. doi:10.1093/toxsci/kfs202. PubMed: 22713597. 22713597

[27] Zhang M, Xuan S, Bouxsein ML, von Stechow D, Akeno N et al. (2002) Osteoblast-specific knockout of the insulin-like growth factor (IGF) receptor gene reveals an essential role of IGF signaling in bone matrix mineralization. J Biol Chem 277: 44005-44012. doi:10.1074/jbc.M208265200. PubMed: 12215457. 12215457

[28] Courtland H-W, Elis S, Wu Y, Sun H, Rosen CJ et al. (2011) Serum IGF-1 Affects Skeletal Acquisition in a Temporal and Compartment-Specific Manner. PLOS ONE 6(3). 10.1371/journal.pone.0014762 PMC3060807 21445249

[29] Tirapegui J, Baldi M, Ribeiro SL (1996) Effect of protein deficiency on plasma insulin-like growth factor-1 (IGF-1) level and protein and proteoglycan synthesis rates in skeletal muscle and bone. Nutrition Research 16: 869-879. doi:10.1016/0271-5317(96)00080-2.

[30] Doyle JJ (1979) Toxic and essential elements in bone--a review. J Anim Sci 49: 482-497. PubMed: 389915. 389915 10.2527/jas1979.492482x

[31] Odstrcil AA, Ricci D, Mandalunis PM (2007) Toxic Effect of Arsenic in Bone Tissue of Experimental Growing Animals. The Preliminary Program for Sociedad Argentina de Investigación Odontológica, Argentine Society of Dental Research Argentina.

[32] Arena JMD (1986) Poisoning. In: Thomas CC 5th ed. Springfield.

[33] Ellenhorn MJ, Barceloux DG (1988) Arsenic In Medical toxicology. Diagnosis and treatment of human poisoning. New York: Elsevier.

[34] Rahman M, Vahter M, Wahed MA, Sohel N, Yunus M et al. (2006) Prevalence of arsenic exposure and skin lesions. A population based survey in Matlab, Bangladesh. J Epidemiol Community Health 60: 242-248. doi:10.1136/jech.2005.040212. PubMed: 16476755. 16476755 PMC2465558

[35] Vahter ME, Li L, Nermell B, Rahman A, El Arifeen S et al. (2006) Arsenic exposure in pregnancy: a population-based study in Matlab, Bangladesh. J Health Popul Nutr 24: 236-245. PubMed: 17195565. 17195565

[36] Ahmed S, Mahabbat-e Khoda S, Rekha RS, Gardner RM, Ameer SS et al. (2011) Arsenic-associated oxidative stress, inflammation, and immune disruption in human placenta and cord blood. Environ Health Perspect 119: 258-264. doi:10.1289/ehp.1102086. PubMed: 20940111. 20940111 PMC3040615

[37] Persson LA, Arifeen S, Ekström EC, Rasmussen KM, Frongillo EA et al. (2012) Effects of prenatal micronutrient and early food supplementation on maternal hemoglobin, birth weight, and infant mortality among children in Bangladesh: the MINIMat randomized trial. JAMA 307: 2050-2059. doi:10.1001/jama.2012.4061. PubMed: 22665104. 22665104

[38] de Onis M, Onyango AW, Borghi E, Siyam A, Nishida C et al. (2007) Development of a WHO growth reference for school-aged children and adolescents. Bull World Health Organ 85: 660-667. doi:10.2471/BLT.07.043497. PubMed: 18026621. 18026621 PMC2636412

[39] Gwatkin DR, Ergo A (2011) Universal health coverage: friend or foe of health equity? Lancet 377: 2160-2161. doi:10.1016/S0140-6736(10)62058-2. PubMed: 21084113. 21084113

[40] Concha G, Vogler G, Lezcano D, Nermell B, Vahter M (1998) Exposure to inorganic arsenic metabolites during early human development. Toxicol Sci 44: 185-190. doi:10.1093/toxsci/44.2.185. PubMed: 9742656. 9742656

[41] Fängström B, Hamadani J, Nermell B, Grandér M, Palm B et al. (2009) Impaired arsenic metabolism in children during weaning. Toxicol Appl Pharmacol 239: 208-214. doi:10.1016/j.taap.2008.12.019. PubMed: 19167415. 19167415

[42] Gardner R, Hamadani J, Grandér M, Tofail F, Nermell B et al. (2011) Persistent exposure to arsenic via drinking water in rural Bangladesh despite major mitigation efforts. Am J Public Health 101 Suppl 1: S333-S338. doi:10.2105/AJPH.2010.300025. PubMed: 21778503. 21778503 PMC3222480

[43] Nermell B, Lindberg AL, Rahman M, Berglund M, Persson LA et al. (2008) Urinary arsenic concentration adjustment factors and malnutrition. Environ Res 106: 212-218. doi:10.1016/j.envres.2007.08.005. PubMed: 17900556. 17900556

[44] Walker BE, Payne RB (1979) Adjusted calcium conflict resolved? Differing effects on plasma total calcium of changes in plasma albumin after venous stasis or myocardial infarction. J Clin Pathol 32: 488-491. doi:10.1136/jcp.32.5.488. PubMed: 469006. 469006 PMC1145712

