# Supplementary material for: Arsenic Exposure Affects Plasma Insulin-Like Growth Factor 1 (IGF-1) in Children in Rural Bangladesh
Source: PLoS One. 2013 Nov 26;8(11):e81530. doi: 10.1371/journal.pone.0081530 (PMC3841153; doi:10.1371/journal.pone.0081530)
Supplement: File S1 — Supporting files. (DOCX) [file pone.0081530.s001.docx]

**File S1**

**Arsenic exposure affects plasma insulin-like growth factor 1 (IGF-1) in children in rural Bangladesh**

Sultan Ahmed^1, 2^, Rokeya Sultana Rekha^1^, Khalid Bin Ahsan^1^, Mariko Doi^3^, Margaretha Grandér^2^, Anjan Kumar Roy^1^, Eva-Charlotte Ekström^4^, Yukiko Wagatsuma^3^, Marie Vahter^2^, Rubhana Raqib^1*^

^1^Centre for Vaccine Sciences, International Centre for Diarrhoeal Disease Research, Bangladesh (icddr,b) , Dhaka, Bangladesh; ^2^Institute of Environmental Medicine (IMM), Karolinska Institutet, Stockholm, Sweden; ^3^Department of Clinical Trial and Clinical Epidemiology, Faculty of Medicine, University of Tsukuba, Tsukuba, Japan; ^4^International Maternal and Child Health, Department of Women’s and Children’s Health, Uppsala University, Uppsala, Sweden.

*Address correspondence to:

Rubhana Raqib, Ph.D.

Nutritional Biochemistry Laboratory , Centre for Vaccine Sciences, International Centre for Diarrhoeal Disease Research, Bangladesh (icddr,b), Dhaka-1212, Bangladesh, Phone- 880-2- 9827001-10, Ext-2404, Fax: +880-29827075 / +880-29827077, Email: [rubhana@icddrb.org](mailto:rubhana@icddrb.org)

**Table S1.** Basic characteristics of the children in 4.5 years of age in relation to IGF-1 (median split), stratified by sex.

| **Variables** | **Boys (N=328)** | | **Girls (N=312)** | |
| --- | --- | --- | --- | --- |
|  | **IGF ≤59 µg/L** | **IGF >59 µg/L** | **IGF ≤59 µg/L** | **IGF >59 µg/L** |
| Height, cm | 100 ± 3.9 | 102 ±3.9* | 98 ± 3.7 | 100 ± 3.6* |
| Weight, kg | 13.9 ± 1.4 | 14.8 ±1.9* | 13.2 ± 1.3 | 13.7 ± 1.5* |
| HAZ | -1.66 (-3.08; -0.16) | -1.16 (-2.53; 0.37)* | -1.89 (-3.27; -0.56) | -1.55 (-2.80; -0.30)* |
| WAZ | -1.83 (-3.15; -0.51) | -1.51 (-2.87; 0.14) | -2.02 (-3.25; -0.79) | -1.75 (-3.01; -0.48) |
| Stunted, n (%) | 67 (36) | 17 (12) | 60 (45) | 44 (24) |
| Wasted, n (%) | 74 (40) | 43 (31) | 69 (51) | 68 (38) |
| U-As, µg/L^a^ | 57 (20; 399) | 58 (20; 305) | 68 (22; 431) | 54 (21; 327)* |
| iAs, % ^b^ | 8.6 ±3.2 | 8.4 ± 2.9 | 8.8 ± 2.9 | 9.1 ± 3.2 |
| DMA, % ^b^ | 81.8 ± 5.2 | 81.6 ± 5.4 | 81.4 ± 5.2 | 80.5 ± 5.4 |
| MMA, % ^b^ | 9.5 ± 3.2 | 9.9 ± 3.6 | 9.8 ± 3.6 | 10.4 ± 3.4 |

Abbreviations: HAZ, height-for-age z-score; WAZ, weight-for-age z-score; U-As; urinary arsenic; iAs, inorganic arsenic; DMA, dimethylarsinic acid; MMA, methylated methylarsonic acid. Data given as mean ± standard deviation, median (5-95 percentiles), or n (%). ^a^Adjusted to average specific gravity of 1.012 g/mL. ^b^Percent of total metabolite concentration in urine. * indicates P<0.05 (Mann-Whitney U) in comparing parameters between above and below median cut-off levels.

**Table S2.** Linear regression analysis of plasma biomarkers in relation to concurrent and prenatal arsenic exposure in all children, boys and girls at 4.5 years age.

| **Variables** | **U-As at 4.5 years (per 10 µg/L)** | | | | | **U-As at GW8 (per 10 µg/L)** | |
| --- | --- | --- | --- | --- | --- | --- | --- |
|  | Unadjusted | | | Adjusted | | Adjusted | |
|  | β (95% CI) | P | | β (95% CI) | P | β (95% CI) | P |
| All children (N=640) | | | | | | | |
| IGF-1, µg/L^a^ | -0.24 (-0.46, -0.006) | 0.04 | -0.27 (-0.49, -0.046) | | 0.01 | 0.0031 (-0.005, 0.012) | 0.49 |
| IGF-1, µg/L^b^ | -0.24 (-0.46, -0.006) | 0.04 | -0.31 (-0.54, -0.069) | | 0.01 | 0.014 (-0.10, 0.13) | 0.81 |
| Adj Ca, mg/dL^a^ | 0.009 (-0.007, 0.027) | 0.25 | 0.006 (-0.012, 0.023) | | 0.50 | 0.0031 (-0.005, 0.012) | 0.49 |
| Vit-D, nmol/L^a^ | 0.098 (-0.029, 0.22) | 0.13 | 0.045 (-0.085, 0.17) | | 0.50 | - 0.0001 (-0.072, 0.071) | 0.99 |
| PTH, ng/L^a^ | -0.010 (-0.13, 0.11) | 0.86 | -0.018 (-0.14, 0.10) | | 0.78 | 0.032 (-0.034, 0.10) | 0.34 |
| B-ALP, µg/L^a^ | 0.098 (-0.19, 0.38) | 0.50 | 0.10 (-0.19¸ 0.40) | | 0.49 | -0.0008 (-0.16, 0.16) | 0.99 |
| PO_4_, mg/dL^a^ | 0.023 (-0.02, 0.06) | 0.30 | 0.025 (-0.019¸ 0.07) | | 0.26 | 0.012 (-0.012, 0.037) | 0.31 |
| Boys (N=328) | | | | | | | |
| IGF-1, µg/L^a^ | -0.21 (-0.58, 0.15) | 0.25 | -0.24 (-0.59, 0.11) | | 0.17 | -0.003 (-0.015, 0.009) | 0.60 |
| IGF-1, µg/L^b^ | -0.21 (-0.58, 0.15) | 0.25 | -0.28 (-0.63, 0.081) | | 0.12 | -0.011 (-0.19, 0.17) | 0.90 |
| Adj Ca, mg/dL^a^ | 0.007 (-0.017, 0.032) | 0.56 | 0.007 (-0.018, 0.032) | | 0.59 | -0.0033 (-0.015, 0.009) | 0.60 |
| Vit-D, nmol/L^a^ | 0.031 (-0.18, 0.25) | 0.77 | -0.003 (-0.22, 0.21) | | 0.98 | 0.047 (-0.066, 0.16) | 0.41 |
| PTH, ng/L^a^ | 0.076 (-0.13, 0.29) | 0.48 | 0.070 (-0.15, 0.28) | | 0.52 | 0.052 (-0.054, 0.15) | 0.33 |
| B-ALP, µg/L^a^ | 0.20 (-0.25, 0.65) | 0.37 | 0.22 (-0.23, 0.67) | | 0.34 | 0.14 (-0.097, 0.37) | 0.25 |
| PO_4_, mg/dL^a^ | 0.08 (0.01, 0.16) | 0.02 | 0.09 (0.011, 0.16) | | 0.02 | 0.029 (-0.011, 0.070) | 0.14 |
| Girls (N=312) | | | | | | | |
| IGF-1, µg/L^a^ | -0.27 (-0.56, 0.01) | 0.05 | -0.30 (-0.59¸ -0.012) | | 0.04 | 0.0069 (-0.006, 0.02) | 0.30 |
| IGF-1, µg/L^b^ | -0.27 (-0.56, 0.01) | 0.05 | -0.32 (-0.63¸ 0.0005) | | 0.05 | 0.023 (-0.13, 0.18) | 0.77 |
| Adj Ca, mg/dL^a^ | 0.011 (-0.011, 0.03) | 0.32 | 0.0007 (-0.023, .025) | | 0.95 | 0.0069 (-0.006, 0.020) | 0.30 |
| Vit-D, nmol/L^a^ | 0.14 (-0.01, 0.29) | 0.06 | 0.069 (-0.089, 0.22) | | 0.38 | -0.050 (-0.14, 0.042) | 0.28 |
| PTH, ng/L^a^ | -0.061 (-0.20, 0.08) | 0.39 | -0.074 (-0.22, 0.07) | | 0.33 | 0.024 (-0.062, 0.11) | 0.58 |
| B-ALP, µg/L^a^ | 0.005 (-0.37, 0.38) | 0.98 | 0.030 (-0.37, 0.43) | | 0.88 | -0.082 (-0.31, 0.14) | 0.48 |
| PO_4_, mg/dL^a^ | -0.016 (-0.065, 0.032) | 0.51 | -0.022 (-0.072¸ 0.028) | | 0.39 | -0.002 (-0.031, 0.027) | 0.87 |

Abbreviations: CI, confidence interval; β, unstandardized regression coefficients; U-As; urinary arsenic ; IGF-1, insulin-like growth factor 1; Adj Ca, albumin adjusted calcium; Vit-D, vitamin D; PTH, parathyroid hormone; B-ALP, bone-specific alkaline phosphatase; PO_4_, phosphate; SES, socioeconomic status. ^a^Adjusted for SES, parity (birth order), child sex (for all children),HAZ and % of monomethyl arsonic acid (MMA). ^b^Adjusted for SES, parity (birth order), child sex (for all children), HAZ, % of MMA and plasma levels of Adj Ca, Vit-D, PTH, B-ALP and PO_4_.

**Table S3**. Linear regression analysis of associations between concurrent arsenic exposure and plasma IGF-1 in 4.5 years old children in relation to growth retardation stratified by % of MMA (median split, 9.7%).

|  | Low % of MMA | | | High % of MMA | | |
| --- | --- | --- | --- | --- | --- | --- |
|  | All children | Boys | Girls | All children | Boys | Girls |
|  | β (95% CI) | β (95% CI) | β (95% CI) | β (95% CI) | β (95% CI) | β (95% CI) |
| Stunted | 0.28 (-0.26, 0.83) | 0.37 (-0.29, 1.04) | 0.24 (-0.84, 1.34) | -0.15 (-0.59, 0.28) | -1.27 (-2.72, 0.18) | -0.049 (-0.60, 0.50) |
| Normal height | -0.21 (-0.77, 0.33) | -0.30 (-1.04, 0.43) | 0.095 (-0.81, 1.0) | -0.44 (-0.82, -0.070) | -0.33 (-0.88, 0.20) | -0.71 (-1.29, -0.13)* |
| Underweight | 0.15 (-0.38, 0.69) | 0.102 (-0.63, 0.84) | 0.17 (-0.69, 1.05) | -0.36 (-0.78, 0.053) | -0.91 (-1.89, 0.070) | -0.25 (-0.76, 0.25) |
| Normal weight | -0.22 (-0.87, 0.42) | -0.33 (-1.20, 0.54) | -0.071 (-1.13, 0.99) | -0.32 (-0.72, 0.065) | -0.18 (-0.76, 0.38) | -.056 (-1.15, 0.018) |

Abbreviations: CI, confidence interval; β, unstandardized regression coefficients; IGF-1, insulin-like growth factor 1. Adjusted for SES, parity (birth order), child sex (for all children), and plasma levels of Adj Ca, Vit-D, PTH, B-ALP and PO_4._ * indicates P<0.05.
